# Supplementary material for: Development and validation of a new prognostic index for mortality risk in multimorbid adults
Source: PLoS One. 2022 Aug 5;17(8):e0271923. doi: 10.1371/journal.pone.0271923 (PMC9355209; doi:10.1371/journal.pone.0271923)
Supplement: S1 Table — (DOCX) [file pone.0271923.s001.docx]

**Supporting Information**

**S1 Table.** Missing data in the predictors in the 805 participants.

| Variable | Missing data, n (%) |
| --- | --- |
| Age | 0 |
| CC-Index^a^ | 0 |
| Drugs | 0 |
| BMI^b^ | 30 (3.7) |
| Hospitalizations | 3 (0.4) |
| Barthel-Index | 9 (1.1) |
| Nursing home residence | 1 (0.1) |

^a^Charlson-Comorbidity-Index

^b^Body-Mass-Index
