# Supplementary material for: Development and validation of a new prognostic index for mortality risk in multimorbid adults
Source: PLoS One. 2022 Aug 5;17(8):e0271923. doi: 10.1371/journal.pone.0271923 (PMC9355209; doi:10.1371/journal.pone.0271923)
Supplement: S2 Table — (DOCX) [file pone.0271923.s002.docx]

**Supporting Information**

**S2 Table.** Univariable analysis of candidate predictors with a Weibull model from information obtained at baseline.

| Variable |  | HR (95% CI) | β coefficient^a^ | p-value |
| --- | --- | --- | --- | --- |
| Age | 70-79 | Ref | Ref |  |
|  | 80-99 | 1.47 (1.19-1.80) | 0.39 | 0.02 |
| Sex | F | Ref |  |  |
|  | M | 0.94 (0.76-1.15) | -0.06 | 0.72 |
| CC-Index^b^ | 0-2 | Ref | Ref |  |
|  | ≥3 | 2.48 (2.06-2.94) | 0.91 | <.001 |
| Drugs | <10 | Ref | Ref |  |
|  | ≥10 | 1.57 (1.29-1.89) | 0.45 | <0.01 |
| BMI | ≥30 | Ref | Ref |  |
|  | <30 | 0.61 (0.39-0.91) | -0.49 | 0.03 |
| Weight loss |  | 1.34 (1.03-1.71) | 0.29 | 0.07 |
| Smoking |  | 1.05 (0.59-1.70) | 0.05 | 0.86 |
| Hospitalizations | 0 | Ref | Ref |  |
|  | >1 | 1.46 (1.19-1.77) | 0.38 | 0.02 |
| Barthel-Index | >90 | Ref | Ref |  |
|  | 61-90 | 0.43 (0.31-0.57) | -0.85 | <0.01 |
|  | 21-60 | 0.31 (0.24-0.41) | -1.16 | <.001 |
|  | <21 | 0.14 (0.11-0.19) | -1.93 | <.001 |
| Falls | 0 | Ref | Ref |  |
|  | 1 | 1.03 (0.71-1.43) | 0.03 | 0.89 |
|  | >1 | 1.58 (1.18-2.07) | 0.46 | 0.01 |
| Nursing home residence |  | 1.07 (0.62-1.71) | 0.07 | 0.79 |

^a^β coefficient=logHR

^b^Charlson-Comorbidity-Index
