# Supplementary material for: Development and validation of a new prognostic index for mortality risk in multimorbid adults
Source: PLoS One. 2022 Aug 5;17(8):e0271923. doi: 10.1371/journal.pone.0271923 (PMC9355209; doi:10.1371/journal.pone.0271923)
Supplement: S3 Table — (DOCX) [file pone.0271923.s003.docx]

**Supporting Information**

**S3 Table.** Multivariable analysis of candidate predictors with a flexible parametric survival model.

| Variable |  | HR (95% CI) | β coefficient | p-value |
| --- | --- | --- | --- | --- |
| Age | 70-79 |  |  |  |
|  | 80-99 | 1.16 (0.92-1.44) | 0.15 | 0.39 |
| Sex |  | 0.92 (0.74-1.13) | -0.08 | 0.63 |
| CC-Index | 0-2 |  |  |  |
|  | ≥3 | 2.42 (2.01-2.89) | 0.88 | <.001 |
| Drugs | <10 |  |  |  |
|  | ≥10 | 1.32 (1.07-1.60) | 0.27 | 0.14 |
| BMI | ≥30 |  |  |  |
|  | <30 | 1.72 (1.43-2.04) | 0.54 | 0.02 |
| Weight loss |  | 1.22 (0.94-1.57) | 0.20 | 0.25 |
| Smoking |  | 0.96 (0.51-1.61) | -0.04 | 0.89 |
| Hospitalizations | 0 |  |  |  |
|  | >1 | 1.17 (0.94-1.43) | 0.16 | 0.40 |
| Barthel-Index | >90 |  |  |  |
|  | 61-90 | 1.93 (1.45-2.50) | 0.65 | <.01 |
|  | 21-60 | 2.67 (1.92-3.60) | 0.98 | <.001 |
|  | <21 | 6.89 (3.79-11.34) | 1.93 | <.001 |
| Falls | 0 |  |  |  |
|  | 1 | 0.77 (0.52-1.10) | -0.26 | 0.25 |
|  | >1 | 1.22 (0.90-1.61) | 0.20 | 0.32 |
| Nursing home residence |  | 1.91 (1.60-2.25) | 0.64 | 0.04 |
