# Supplementary material for: Development and validation of a new prognostic index for mortality risk in multimorbid adults
Source: PLoS One. 2022 Aug 5;17(8):e0271923. doi: 10.1371/journal.pone.0271923 (PMC9355209; doi:10.1371/journal.pone.0271923)
Supplement: S4 Table — (DOCX) [file pone.0271923.s004.docx]

**Supporting Information**

**S4 Table.** Univariable analysis of the specific items in the Charlson-Comorbidity-Index and Barthel-Index.

| Variable |  | HR (95% CI) | β coefficient | p-value |
| --- | --- | --- | --- | --- |
| Myocardial infarction |  | 1.19 (0.65-1.95) | 0.17 | 0.55 |
| Congestive heart failure |  | 1.58 (1.22-1.99) | 0.46 | <0.01 |
| Peripheral vascular disease |  | 1.11 (0.81-1.47) | 0.10 | 0.57 |
| Cerebrovascular disease |  | 0.96 (0.73-1.24) | -0.04 | 0.81 |
| Dementia |  | 0.90 (0.53-1.42) | -0.10 | 0.69 |
| COPD |  | 1.51 (1.12-1.97) | 0.41 | 0.02 |
| Rheumatoid disease |  | 1.82 (1.09-2.83) | 0.60 | 0.02 |
| Peptic ulcer disease |  | 1.26 (0.54-2.43) | 0.23 | 0.56 |
| Mild liver disease |  | 2.18 (1.38-3.26) | 0.78 | <.001 |
| Hemi/paraplegia |  | 1.21 (0.67-1.99) | 0.19 | 0.51 |
| Renal disease |  | 1.29 (1.02-1.61) | 0.25 | 0.11 |
| Cancer |  | 1.59 (1.21-2.04) | 0.47 | <0.01 |
| Moderate or severe liver disease |  | 1.79 (0.44-4.63) | 0.58 | 0.32 |
| Metastatic solid tumour |  | 3.47 (2.26-5.05) | 1.24 | <.001 |
| Bathing |  | 0.31 (0.23-0.39) | -1.18 | <.001 |
| Bladder | 0 |  |  |  |
|  | 5 | 0.47 (0.31-0.68) | -0.75 | <0.01 |
|  | 10 | 0.52 (0.42-0.63) | -0.66 | <.001 |
| Bowels | 0 |  |  |  |
|  | 5 | 0.77 (0.47-1.12) | -0.26 | 0.44 |
|  | 10 | 0.34 (0.28-0.40) | -1.08 | <.001 |
| Dressing | 0 |  |  |  |
|  | 5 | 0.49 (0.36-0.64) | -0.71 | <.001 |
|  | 10 | 0.27 (0.21-0.34) | -1.29 | <.001 |
| Feeding | 0 |  |  |  |
|  | 5 | 0.30 (0.18-0.48) | -1.19 | <0.01 |
|  | 10 | 0.18 (0.15-0.21) | -1.74 | <.001 |
| Grooming |  | 0.36 (0.29-0.44) | -1.02 | <.001 |
| Mobility | 0 |  |  |  |
|  | 5 | 0.69 (0.27-1.39) | -0.38 | 0.39 |
|  | 10 | 0.66 (0.45-0.93) | -0.41 | 0.08 |
|  | 15 | 0.32 (0.26-0.39) | -1.13 | <.001 |
| Stairs | 0 |  |  |  |
|  | 5 | 0.53 (0.31-0.83) | -0.64 | 0.02 |
|  | 10 | 0.36 (0.28-0.46) | -1.01 | <.001 |
| Toilet use | 0 |  |  |  |
|  | 5 | 0.62 (0.43-0.85) | -0.48 | 0.04 |
|  | 10 | 0.30 (0.24-0.37) | -1.19 | <.001 |
| Transfer | 0 |  |  |  |
|  | 5 | 0.43 (0.25-0.69) | -0.84 | 0.01 |
|  | 10 | 0.42 (0.24-0.67) | -0.86 | <0.01 |
|  | 15 | 0.28 (0.29-0.34) | -1.27 | <.001 |
