# Supplementary material for: Development and validation of a new prognostic index for mortality risk in multimorbid adults
Source: PLoS One. 2022 Aug 5;17(8):e0271923. doi: 10.1371/journal.pone.0271923 (PMC9355209; doi:10.1371/journal.pone.0271923)
Supplement: S5 Table. A. Stratification of 1-year mortality risk by points of the final score. B. Stratification of 1-year mortality risk by points of the simplified score — (DOCX) [file pone.0271923.s005.docx]

**Supporting Information**

**S5A Table.** Stratification of 1-year mortality risk by points of the final score.

| Point score | No. who died / No. at risk (%) | No. who died / No. at risk (%) | Risk category |
| --- | --- | --- | --- |
| 0 | 0 | 16 / 246 (7) | Low |
| 1 | 0 |  |  |
| 2 | 4 / 52 (8) |  |  |
| 3 | 6 / 93 (6) |  |  |
| 4 | 5 / 70 (7) |  |  |
| 5 | 1 / 31 (3) |  |  |
| 6 | 10 / 59 (17) | 66 / 353 (19) | Moderate |
| 7 | 18 / 116 (16) |  |  |
| 8 | 15 / 87 (17) |  |  |
| 9 | 14 / 60 (23) |  |  |
| 10 | 9 / 31 (29) |  |  |
| 11 | 19 / 58 (33) | 76 / 206 (37) | High |
| 12 | 13 / 46 (28) |  |  |
| 13 | 18 / 39 (46) |  |  |
| 14 | 11 / 23 (48) |  |  |
| 15 | 2 / 7 (29) |  |  |
| 16 | 4 / 13 (31) |  |  |
| 17 | 4 / 12 (33) |  |  |
| 18 | 1 / 2 (50) |  |  |
| 19 | 1 / 2 (50) |  |  |
| 20 | 2 / 3 (67) |  |  |
| 21 | 1 / 1 (100) |  |  |

**S5B Table.** Stratification of 1-year mortality risk by points of the simplified score.

| Point score | No. who died / No. at risk (%) | No. who died / No. at risk (%) | Risk category |
| --- | --- | --- | --- |
| 0 | 0 | 45 / 352 (13) | Low |
| 1 | 0 |  |  |
| 2 | 0 |  |  |
| 3 | 0 |  |  |
| 4 | 15 / 116 (13) |  |  |
| 5 | 0 / 2 (0) |  |  |
| 6 | 0 |  |  |
| 7 | 30 / 234 (13) |  |  |
| 8 | 4 / 14 (29) | 113 / 453 (25) | High |
| 9 | 0 |  |  |
| 10 | 66 / 283 (23) |  |  |
| 11 | 8 / 34 (24) |  |  |
| 12 | 0 |  |  |
| 13 | 32 / 102 (31) |  |  |
| 14 | 3 / 20 (15) |  |  |
