# Supplementary figures and images for: Development and validation of a new prognostic index for mortality risk in multimorbid adults
Source: PLoS One. 2022 Aug 5;17(8):e0271923. doi: 10.1371/journal.pone.0271923 (PMC9355209; doi:10.1371/journal.pone.0271923)

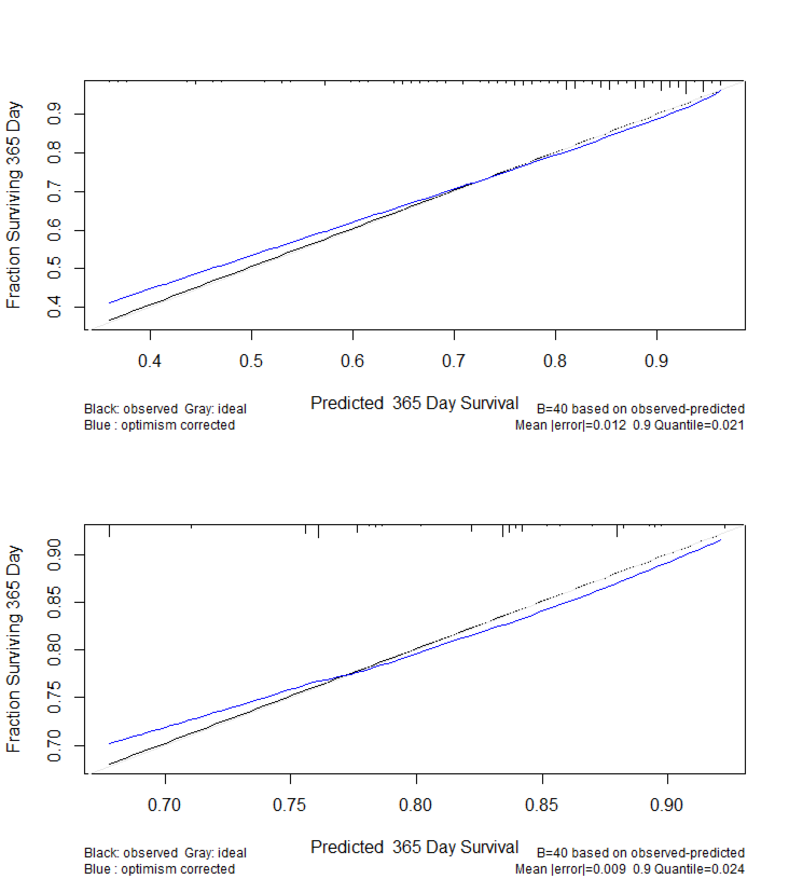

Supplement: S1 Fig — A. Calibration plot of the final prediction model for 1-year mortality. B. Calibration plot of the simplified model for 1-year mortality. (TIF) [file pone.0271923.s006.tif]
